# Large area fractional laser treatment of mouse skin increases energy expenditure

**DOI:** 10.1016/j.isci.2023.108677

**Published:** 2023-12-07

**Authors:** Nunciada Salma, Michael Wang-Evers, Daniel Karasik, Armen Yerevanian, Heather Downs, Tuanlian Luo, Abigail E. Doyle, Zeina Tannous, Jose M. Cacicedo, Dieter Manstein

**Affiliations:** 1 Cutaneous Biology Research Center, Massachusetts General Hospital, Boston, MA 02129, USA; 2 Technion - Israel Institute of Technology, Ruth and Bruce Rappaport Faculty of Medicine, Haifa, Israel; 3 Department of Medicine, Diabetes Unit, Endocrine Division, and Center for Genomic Medicine, Massachusetts General Hospital, Boston, MA 02114, USA; 4 Department of Dermatology, School of Medicine, Lebanese American University, Beirut, Lebanon; 5 Wellman Center for Photomedicine, Massachusetts General Hospital, Boston, MA 02114, USA; 6 Department of Research and Development ALPCO Diagnostics, Salem, NH, USA

**Keywords:** Immunology, Dermatology, Photomedicine, Biological sciences

## Abstract

Fractional laser (FL) treatment is a common dermatologic procedure that generates arrays of microscopic treatment zones separated by intact tissue, promoting fast wound healing. Using a mouse model, we introduced a large area fractional laser treatment (LAFLT) method to study metabolic effects. Using two laser modalities, ablative FL (AFL) and non-ablative FL (NAFL), and exposing different percentages of mice’s total body surface area (TBSA), we followed changes in metabolic parameters in real time using metabolic cages. Additionally, body composition, markers of inflammation, neurohormonal signaling, and browning of adipocytes were investigated. LAFLT, especially in high TBSA groups, had specific metabolic effects such as significantly increased average daily energy expenditure, increased fat mass loss, systemic browning of adipocytes, and inflammatory states, without compromising other organs. The ability of LAFLT to stimulate metabolism in a controlled way could develop into a promising therapeutic treatment to induce positive metabolic changes that replace or augment systemic drugs.

## Highlights

• Localized laser skin treatment resulted in systemic metabolic effects in mice
• Treated mice increased energy expenditure up to 91% over a 6-day period
• Browning of fat, heightened fat use, and short-term inflammation occurred
• More extensive laser coverage amplified the metabolic impact and promoted fat loss

## Graphical abstract

**Figure:**
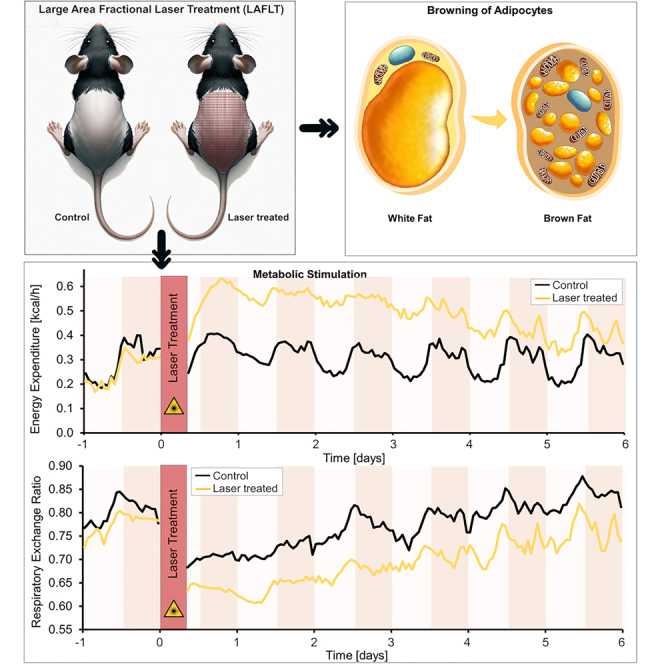

## Introduction

In the past two decades, rates of excess weight and obesity have reached epidemic proportions in developed countries and present a major public health challenge. For many overweight and obese individuals, behavioral and lifestyle modifications are insufficient for long-term weight loss maintenance. Surgical procedures and/or pharmacological interventions that alter either appetite or absorption of food are often pursued as alternative treatment modalities.^1^ Weight-reducing medication and surgical weight loss procedures, however, are expensive and can present significant physical and mental health related complications and side effects.^2^^,^^3^^,^^4^ Therefore, it is not surprising that there is an increasing demand for alternative fat removal procedures, especially for patients who are not candidates for surgical or pharmacological interventions. Here, we introduce large area fractional laser treatments (LAFLT) as a means to stimulate metabolism by systemically increasing energy expenditure through the subsequent inflammatory and healing process following fractional laser (FL) skin treatment. FL treatments consist of the delivery of highly focused short laser pulses to produce arrays of microscopic treatment zones (MTZ), resulting in controlled thermal injury to the skin.^5^^,^^6^^,^^7^ Due to the small diameter of Individual MTZs in the range of 100–300 μm, they allow for fast wound healing by the repair capability of the undamaged surrounding skin.^8^ Two FL treatment modalities, ablative (AFL) and non-ablative (NAFL), have been established for cutaneous remodeling purposes and to improve the appearance of photodamaged and aging skin.^6^^,^^9^ AFL utilizes a highly absorbed laser, typically in the 2000–11000 nm wavelength range such as focused Er:YAG or CO_2_ laser beams, resulting in a small drill of physically vaporized tissue surrounded by a small coagulation cuff. NAFL utilizes a moderately absorbed laser, typically a focused laser beam in the 1000–2000 nm wavelength range, resulting in a small cone-shaped thermal coagulation without the removal of tissue.^5^^,^^10^ A typical FL treatment area of the human face, neck, and décolleté covers around 10% of the total body surface area (TBSA) and, due to the fractional pattern with a fill factor of up to 15%, results in approximately 1.5% TBSA thermal injury. Therefore, there is a clear distinction between the treatment area (i.e., 10% TBSA) and the net coverage rate of thermal injury (i.e., 1.5% TBSA thermal injury). To date, no metabolic changes have been reported or investigated in response to clinically applied FL treatments. However, classical confluent traumatic burn injuries that affect about 20% or more of the TBSA are known to induce a persistent hypermetabolic stress response with systemic dysregulation.^11^^,^^12^^,^^13^ Immediately after a serious burn, sympathetic activation, through adrenergic stimulation, mediates a broad inflammatory response to promote wound healing.^12^ If the systemic inflammatory response continues, a hypermetabolic state follows, which is characterized by a prolonged elevation in oxygen consumption and basal metabolic rate (BMR) with or without accompanying hyperglycemia, loss of lean and fat mass, increased levels of stress mediators and inflammatory cytokines, insulin resistance, browning of subcutaneous fat, and enlargement of the liver and spleen.^11^^,^^12^^,^^14^^,^^15^^,^^16^ It is estimated that half of this elevated BMR relates to wound healing and thermoregulation, and the other half of prolonged high BMR in time occurs due to the upregulation of damaging pathways linked to muscle catabolism.^12^^,^^17^ Initially, this multifactorial response is beneficial to meet the heightened energy demands of the burn condition, however, chronic hypermetabolism can last for up to 24 months and results in significant organ dysfunction, hepatic steatosis, muscle wasting, stress-induced diabetes, lipolysis, and immunosuppression.^18^^,^^19^ Treatment of large area burn injuries therefore includes increasing caloric intake.

Here, we hypothesized that large FL injuries (coverage rate of thermal injury >1.6% TBSA) could induce a controlled and scalable metabolic response that temporarily increases energy expenditure and reduces body weight. Therefore, we developed a mouse model to create a laser skin treatment that produces a large, fast-healing burn injury and a controlled metabolic response without causing chronic hypermetabolism. Our results indicate that a systemic increase in energy expenditure, total mass change, fat utilization, and adipocyte browning can be achieved by FL treatment applied to a large area. LAFLT serves as a promising therapeutic tool to increase energy expenditure and induce weight loss and positive metabolic changes in the overweight population.

## Results

### Metabolic response to LAFLT

**Figure 1:**
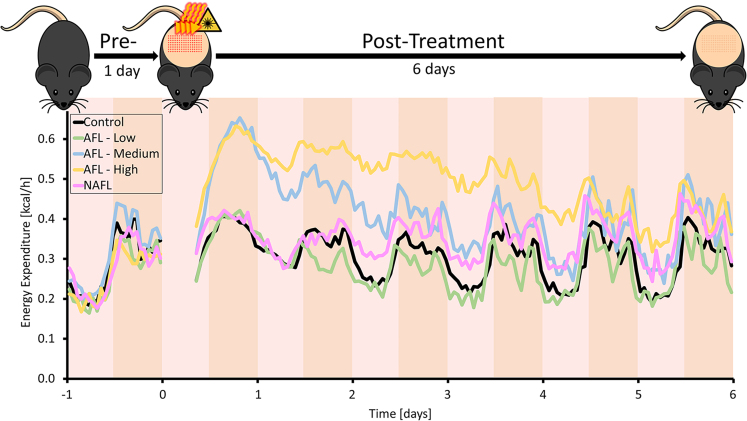
Metabolic cage data and treatment timeline After a 2-day acclimation period, baseline metabolic data were collected for 1 day. On the treatment day, mice were anesthetized, shaved, depilated, treated with an AFL or NAFL, and returned to the metabolic cages. 6 days after the treatment, mice were euthanized for tissue and organ collection. See also Table S1.

**Figure 2:**
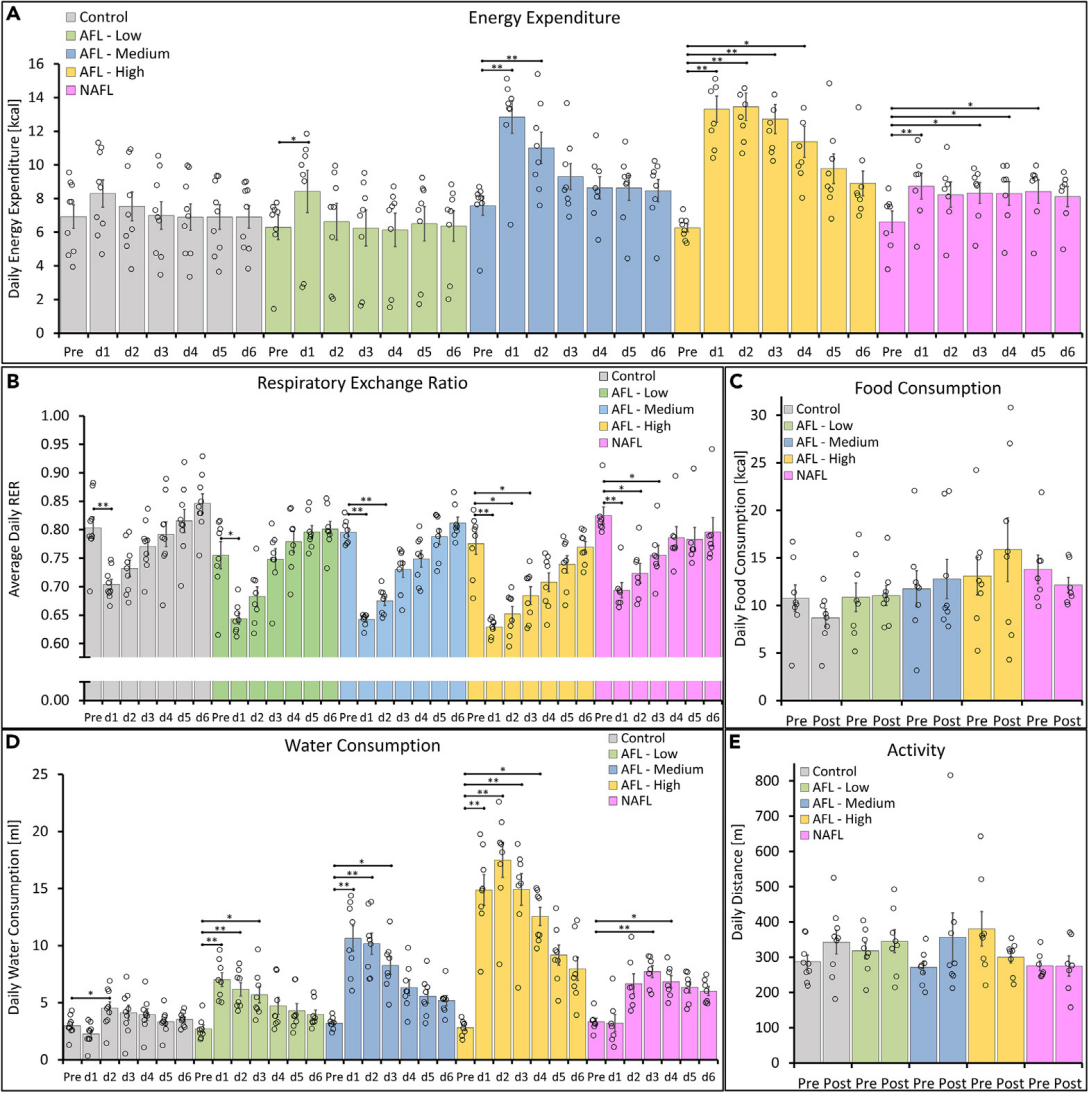
Metabolic cage analysis pre- and post-LAFLT (A–E) Average daily (A) energy expenditure, (B) respiratory exchange rate, (C) food consumption, (D) water consumption, and (E) Locomotor activity. All data are presented as means ± standard deviation. Differences were calculated using a non-parametric repeated measures ANOVA. p∗<0.05, p∗∗<0.001.

A series of metabolic measurements were collected in two periods: 1 day pre- and 6 days post-FL treatment (Figure 1). The metabolic data was used to analyze the daily EE, respiratory exchange ratio, water consumption, food consumption, and locomotor activity. Figures 1 and 2A show a substantial EE increase during the first 3–5 days post treatment for the AFL-Medium, AFL-High, and NAFL groups. EE for NAFL group was elevated and stable compared to the AFL treatments. There was a mild increase in EE for the AFL-Low, compared to the control group. Post-FL treatment, the EE over a 6-day period increased by 5% for control, 5% for AFL-Low, 29% for AFL-Medium (p < 0.05), 91% for AFL-High (p < 0.001), and 30% for NAFL (p < 0.001) groups. Depending on the group, this translates to an additional post treatment EE of 2–34 kcal (8–142 kJ) over a 6-day period. As expected, treatment settings that correspond to current clinical applications of the face and neck (AFL-Low, coverage rate of thermal injury 1.6% TBSA) did not result in a lasting increase in EE. However, increasing the coverage rate of thermal injury to 3.2%, 4%, and 8% TBSA resulted in a significant EE increase post treatment for the AFL-Medium, NAFL, and AFL-High groups, respectively.

We analyzed the respiratory exchange ratio (RER) to assess energy fuel utilization (Figure 2B). We observed that both the treatment and control groups caused a significant reduction in RER which normalized over 6 days. Changes in RER in the control group could be explained by shaving, depilation, and anesthesia. The short-term effect of ketamine, reduced insulation due to the loss of fur, and consequent body heat loss, even in a thermo-neutral environment, contributed to this increase in RER. Stress and dermal inflammation due to shaving also contributed to this. Mice receiving FL treatment had significantly decreased RER over time when compared to the control group. These effects are significant in all FL-treated groups, although more noticeable in AFL-Medium and AFL-High groups. This indicates that FL treatment is directly promoting lipid mobilization and beta-oxidation.

The FL injuries impair the epidermal barrier and increase capillary permeability, resulting in significant fluid loss via water evaporation.^20^^,^^21^ Therefore, it is not surprising that the water consumption increased significantly (p < 0.001) for all treatment groups (Figure 2D) in a dose-dependent manner. For the daily consumption of food and the average daily distance covered in the cages, the results show no significant changes pre- and post-FL treatment for all groups (Figures 2C and 2E).

### Body composition changes after LAFLT

**Figure 3:**
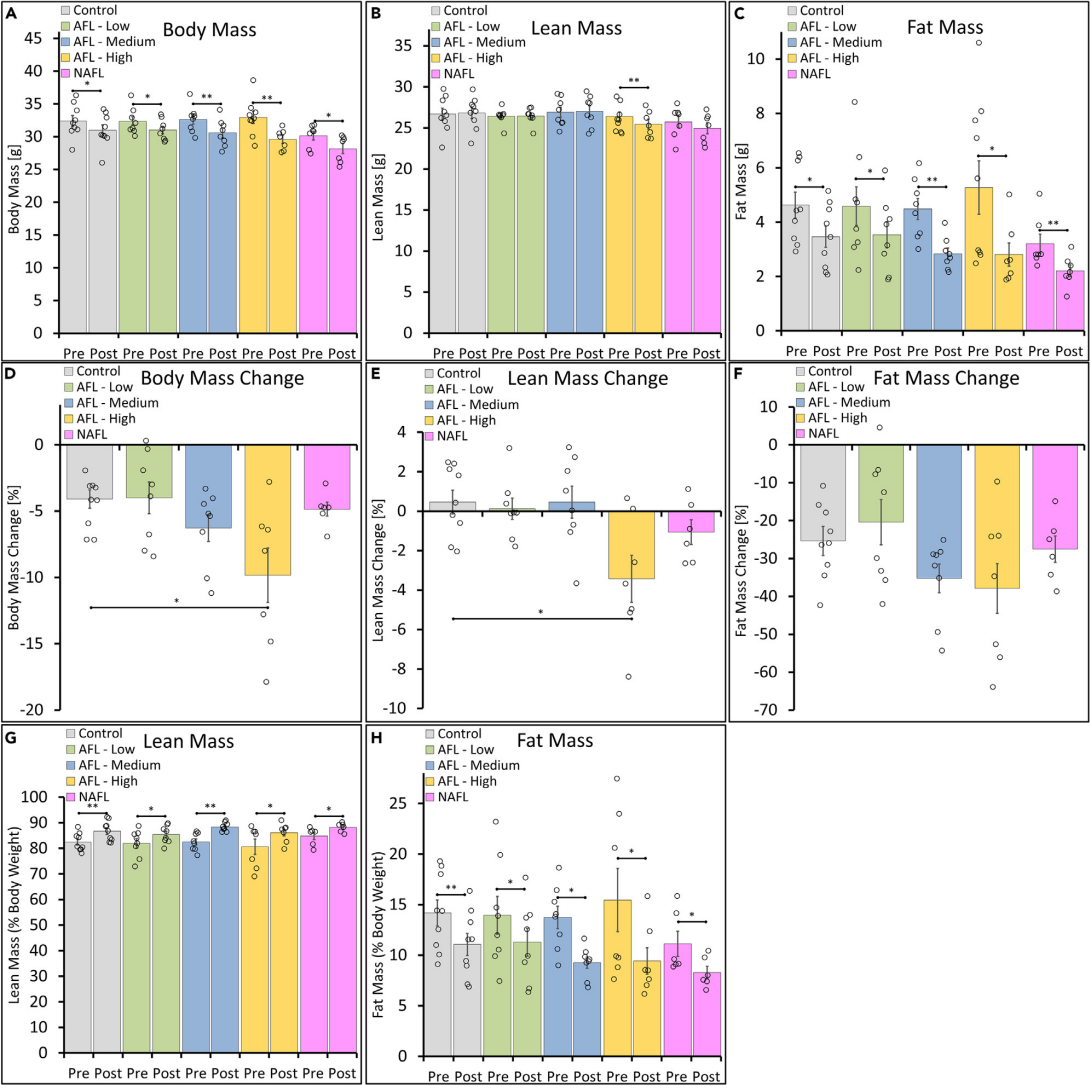
Body Composition changes after LAFLT (A–H) Body composition analysis pre and 6 days post-LAFLT showing the change in (A) body mass, (B) lean mass, and (C) fat mass. The (D) body, (E) lean, and (F) fat mass change pre and 6 days post-FL treatment. The (G) lean mass and (H) fat mass is expressed as % of the body weight. The control group received the same pretreatments (shaving, depilation, buprenorphine and ketamine/xylazine anesthesia) as the fractional laser treatment groups. Data are represented as mean ± SEM. Differences were calculated using t-test. p∗<0.05, p∗∗<0.001.

The body composition analysis at 6 days post-FL treatment resulted in a significant weight loss for the control and laser-treated groups (Figure 3A) ranging from 4–9.8%. Compared to the control group, the weight change was significant for the AFL-High group (p = 0.014) (Figure 3D). Concurrently there was a reduction in lean mass for the AFL-High treatment group (Figures 3B and 3E), similarly to the lean mass loss caused by severe burn wounds as part of the hypermetabolism cascade.^15^ The analysis of fat mass showed a significant reduction for all treatment groups (Figure 3C). Compared to 25% fat mass loss for the control group, LAFLT caused a more remarkable fat loss of 35% and 38%, particularly when larger areas of skin were treated for AFL-Medium and AFL-High, respectively (Figure 3F).

### Skin histology after LAFLT

**Figure 4:**
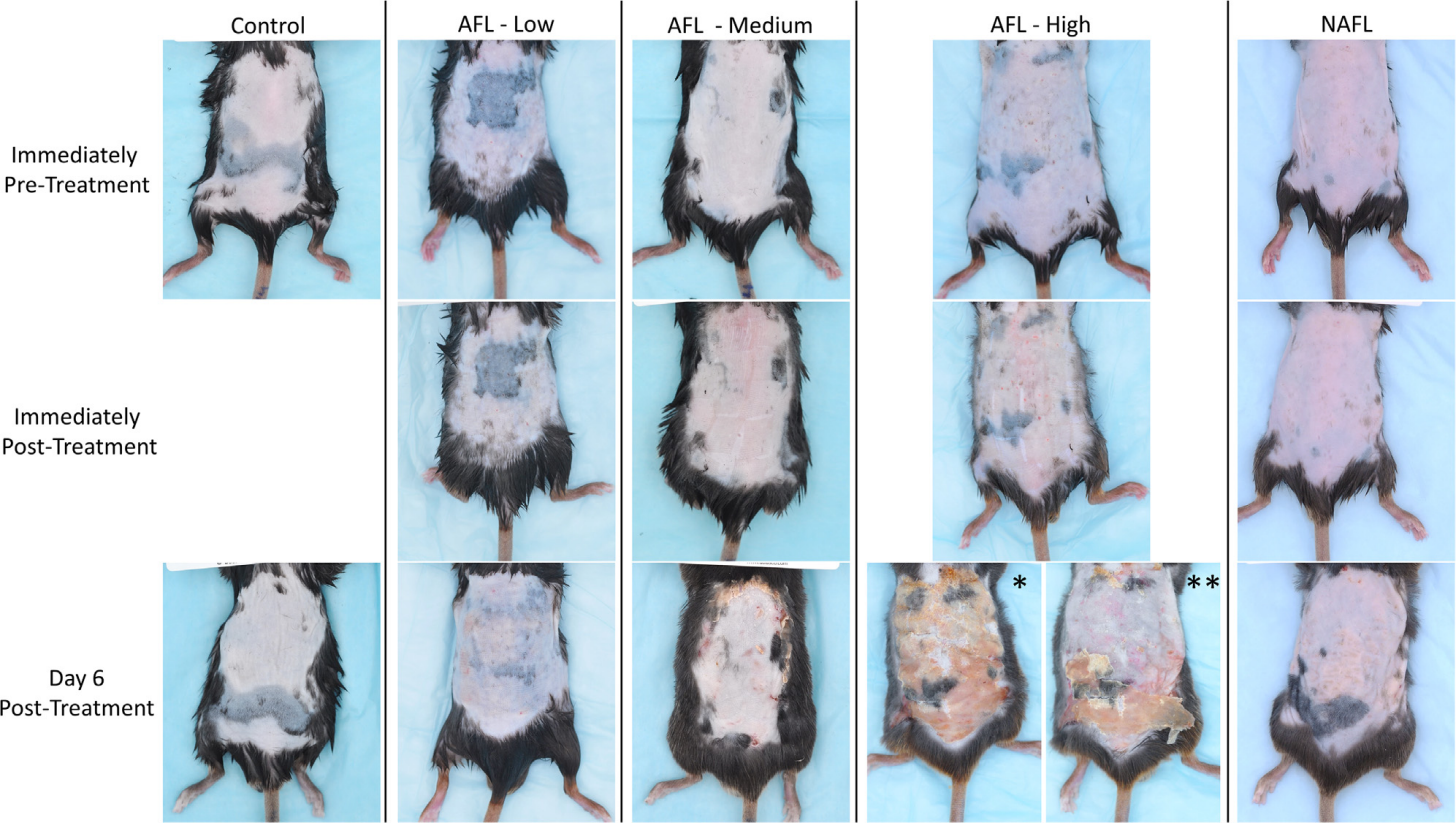
Skin changes after LAFLT Photographs of mice showing the surface healing response following LAFLT. The top row of photographs shows representative mice before FL treatment, the center row shows the same mice immediately after laser exposure, and the bottom row shows them 6 days post exposure. Immediately after laser treatment, a skin tightening effect could be observed. On day 6 post treatment, all laser thermal injuries healed scar-free, however, the AFL-High mice showed scabbing (∗), which was easily removed (∗∗), and presented an underneath intact, healthy skin with light erythema.

**Figure 5:**
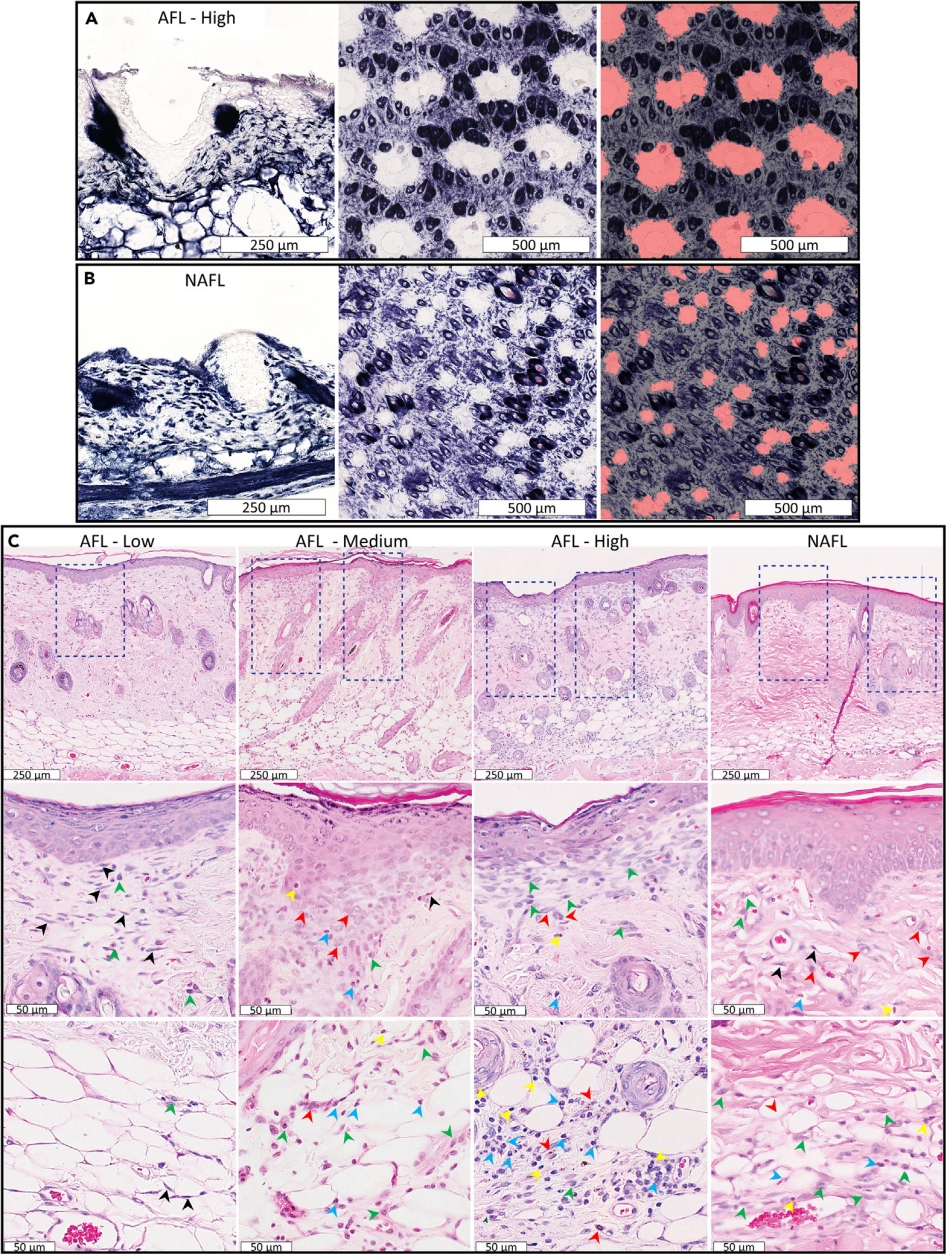
Histological changes of skin immediately and after 6 days of LAFLT The NBTC stain was used to highlight thermally injured tissue (loss of blue stain) immediately after laser exposure for: (A) Vertical and horizontal sections of a mouse skin treated with an AFL with energies of 17 mJ/pulse and 25% density as determined by the segmentation of thermally injured tissue. (B) Vertical and horizontal sections of mouse skin treated with a NAFL with energies of 8 mJ/pulse, 7 passes at 2.2% fractional density resulting in 15% fractional density as confirmed by the segmentation of the thermally injured tissue. (C) H&E histology 6 days post-LAFLT. Laser-induced lesions are outlined, and immune cell infiltrate is presented in higher magnification. Black arrows show fibroblasts, blue arrows show neutrophils, green arrows show histiocytes, yellow arrows show lymphocytes, and red arrows show foaming macrophages.

In order to evaluate wound healing and histologic changes induced by AFL and NAFL, we assessed the thermal damage of the treated mice skin after FL treatment (Figures 4 and 5). Figures 5A and 5B shows nitro blue tetrazolium chloride (NBTC)-stained histological sections of mice skin immediately after laser treatments. Photothermal lesions produced by AFL (Figure 5A) and NAFL (Figure 5B) are shown. The typical cone-shaped epidermal-dermal disruption with coagulation of collagen from both lasers is evident immediately after FL treatment. Comparing the two lesion types, the AFL laser settings generated deeper and wider lesions at 220 μm depth and 200 μm width (ablation + CZ). The NAFL lesions are shallower and narrower at 170 μm depth and 70 μm width. As expected, AFL lesions show a loss of dermal and epidermal tissue, while NAFL lesions show thermal damage of the dermis and epidermis with no tissue loss. These differences in lesion characteristics have an impact on wound healing since smaller NAFL lesions heal faster compared to larger AFL wounds (Figures 4 and 5C). Additionally, we verified histologically that the NAFL lesions resulted in a 15% fractional area coverage and that the AFL lesions resulted in a 16% and 25% fractional area coverage for the AFL-Low/Medium and AFL-High settings, respectively (Figures 5A and 5B). FL treatment triggered a wound-healing response characterized by an infiltration of immune cells. This response has been described in mice, pigs, and humans.^6^^,^^22^ On day 6 after LAFLT, re-epithelization and dermal wound healing were seen in most FL-treated mice. Many inflammatory cells, including histiocytes, neutrophils, lymphocytes, and monocytes, are present in the treated areas (Figure 5C). The wound-healing process is near completion for the MTZs of AFL-Low and NAFL groups, while it is still ongoing for the AFL-Medium and AFL-High treatments due to the presence of numerous neutrophils. Myofibroblasts involved in forming extracellular matrix and in wound contraction are present at the MTZs of AFL-Low, AFL-Medium, and NAFL groups, while foam macrophages are present for AFL-Medium, AFL-High, and NAFL treatments.^23^ Collagen deposition is not evident for any FL-treated mice on day 6 (Figure 5C). Histiocytes are present for all treatment groups, and lymphocytes were detected for AFL-Medium, AFL-High, and NAFL groups. The epidermis in all LAFLT groups exhibits dyskeratotic keratinocytes, and spongiosis is seen in the NAFL-treated group, which is distinctive for NAFL treatments. The evaluation of the entire wound healing process until normalization will require future time course assessments. We also observed an infiltrate of immune cells at the subcutaneous layer of the skin in AFL-Medium and High, as well as in the NAFL-treated mice indicating that inflammation was present in the subcutaneous fat after FL treatments for those laser settings. This is not surprising considering that the skin of mice is thin and the treated TBSA for these laser settings was high. This contributed to the triggering or mobilizing of immune cells to subcutaneous fat.

### LAFLT induces browning of white adipocytes

Induction of adipocyte browning by FL treatment has not been previously studied. We examined the presence of uncoupling protein 1 (Ucp1), a marker of adipocyte browning, and a well-known protein involved in non-shivering thermogenesis in several fat pads (Figure S1A). Ucp1-positive cells were found in all fat pads of FL-treated groups, especially in the AFL-High and NAFL-treated mice implying that FL treatment induced browning. Multilocular adipocytes, a morphological characteristic of beige adipocytes, were found in all FL-treated mice, although they were more prominent in AFL-Medium, AFL-High, and NAFL-groups. The analysis of adipocyte size showed that the fat pads of FL-treated mice contained significantly smaller adipocytes than the control group (Figure S1B). Our results indicate that white adipocyte tissue is shifted to a more thermogenic phenotype in response to FL treatment.

### Cytokine and catecholamine response after LAFLT

**Figure 6:**
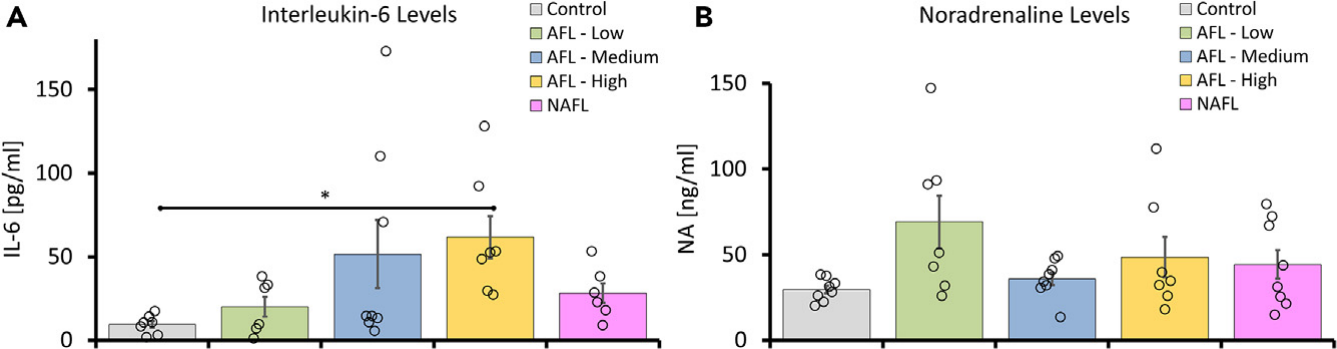
Cytokine and catecholamine response after LAFLT IL-6 and NA levels in FL-treated mice vs. control were determined on day 6. (A) IL-6 levels from AFL-High were statistically significant compared to sham groups (p = 0.003, one-way ANOVA). (B) The NA concentrations in all FL groups were not statistically significant compared to the sham group (p = 0.0784, one-way ANOVA). Data are represented as mean ± SEM. See also Figure S1.

Even though LAFLT produces microscopic thermal lesions, the density and distribution over a large area of the skin triggers a systemic inflammatory response. Two main aspects of inflammation that occur after a burn are considered in our study. The primary aspect involves the immune infiltrate response that occurs during wound healing, which is stimulated by skin damage induced by the laser. A second aspect refers to the measurement of IL-6, one of the most evaluated cytokines in mouse models of burn, because it plays a central role in the inflammatory response to burn injury. Il-6 is particularly important to measure because of its central role in the inflammatory response and its involvement in complications of burn injury, mediating cellular repair and control of homeostasis. This inflammatory response was assessed by histological evaluation of the skin (Figure 5) and by blood levels of a burn-linked cytokine (IL-6) and the stress hormone noradrenaline (NA, Figure 6). Levels of IL-6, were elevated on day 6 post treatment in all groups compared to the control (Figure 6A) and correlated to the thermal injury severity (Figure 4). This was expected since the skin post-FL treatment showed a certain degree of erythema for the AFL-Medium, AFL-High group (significant levels of IL 6), and NAFL groups. Catecholamines such as NA are critical mediators of the metabolic response in adipose tissue via adrenergic receptors.^24^ FL treatments resulted in thermal injury (coverage rate of thermal injury of 1.6%–8% TBSA) that stimulated the release of NA to regulate wound healing. While the NA concentrations 6 days post treatment tended to be higher in the FL-treated groups compared to the control group, the increases were not statistically significant (p = 0.078) (Figure 6B). However, based on previous experiments (data not shown), we expect NA to be significantly elevated up to 4 days post treatment. The NA stress triggered by the FL treatment is sufficiently long to stimulate the desired effects of wound healing and browning of adipocytes and short enough, we speculate, to avoid causing long-lasting hypermetabolism.

### Organ analysis

**Figure 7:**
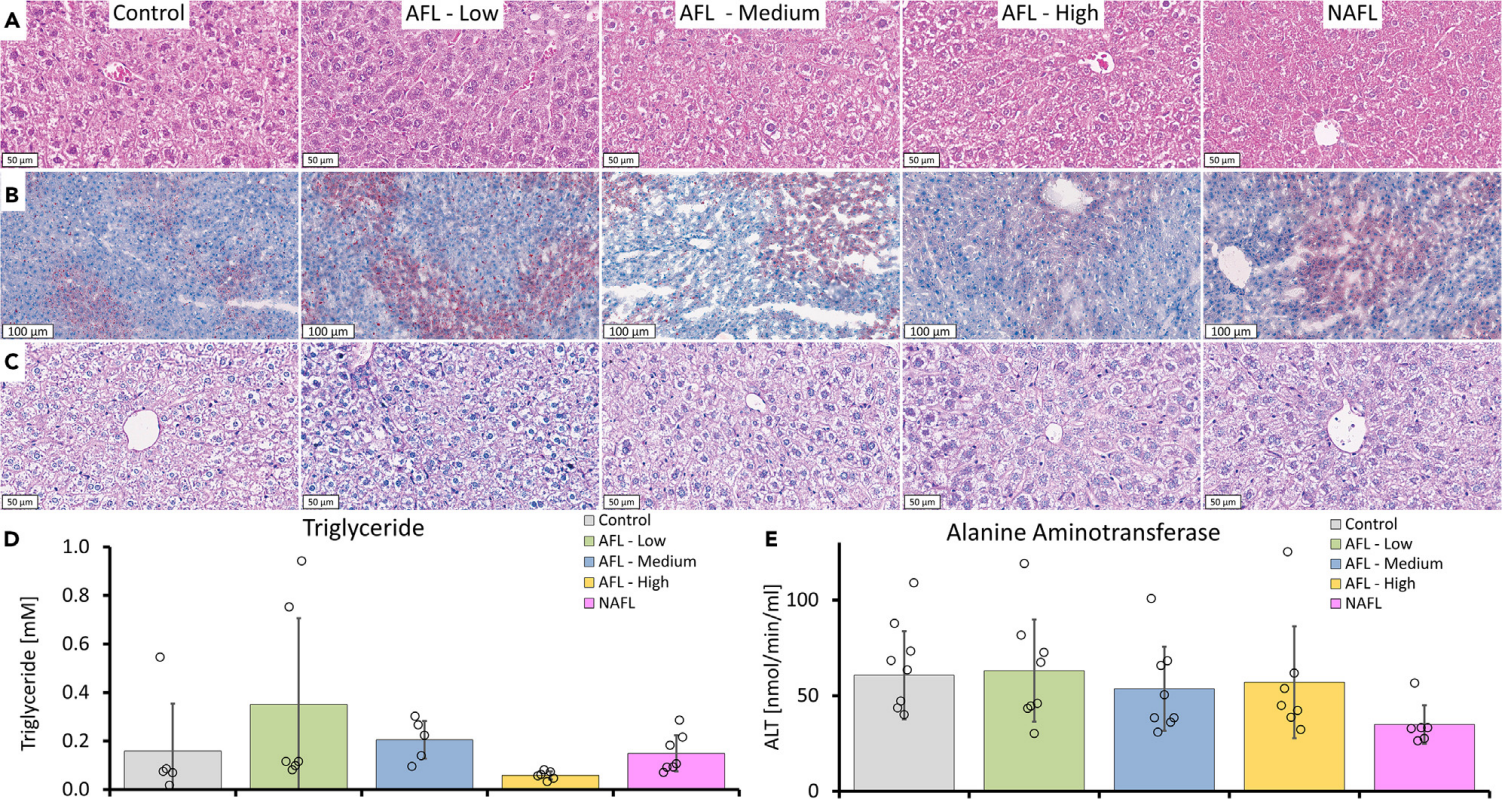
Histological evaluation of livers on day 6 after LAFLT (A) H&E, (B) Oil red O, and (C) periodic acid Schiff stains. (D) Quantitative analysis of TG content of livers. (E) Quantitative analysis of ALT activity. See also Figures S2 and S3.

Large burn injuries (>10% TBSA) can trigger a hypermetabolic response that is linked to changes in organ function and metabolic requirements.^15^ Even though FL treatment in our study induced fractional thermal injuries that ranged from a coverage rate of 1.6%–8% TBSA, systemic effects were observed on day 6 post-LAFLT. Browning of white adipose tissue (WAT) is linked to an increase in lipolysis and could induce hepatic steatosis after a severe burn injury. To evaluate this possible outcome, livers from all mice groups were stained with H&E staining (Figure 7A). The cytoplasm of the hepatocytes from all groups contained vacuoles. To discern the lipid content, we used Oil red O (ORO) stain and total triglyceride (TG) quantification. Interestingly, ORO staining showed minimal lipid accumulation after LAFLT (Figure 7B). Quantitative TG determination showed no significant differences (p = 0.2007) in TG content of the livers of FL-treated mice compared to the control group (Figure 7D), which validated the ORO staining results. As expected, the trends in TG quantity and ORO staining are similar (Figures 7B and 7D). Our results showed that thermal injuries produced by LAFLT did not result in significant lipid alteration in the liver at the time of evaluation. To determine glycogen content, a periodic acid Schiff (PAS) stain with digestion was performed. The PAS stain showed that the vacuoles are caused by glycogen accumulation (Figure 7C) which was consistent throughout all groups and caused by the feeding state of the mice at the time of evaluation (Figure S3). Together, these results indicate that LAFLT does not induce hepatic steatosis and does not increase glycogen accumulation. While the liver weight and size have not been quantitively analyzed, we did not observe, during dissection, an evident enlargement of the livers post treatment. This is supported by our analysis of the liver histology, which did not show signs of hepatocyte hyperplasia, which would have shown an increased number of cells with double nuclei (Figure 7A). Interestingly, after a severe burn injury, hyperplasia of the liver is a common and reversible finding that correlates to the severity of the thermal injury.^13^^,^^14^ To assess hepatocyte injury after LAFLT, alanine aminotransferase (ALT) levels were determined. Levels of ALT were normal in all FL treatment groups compared to the control group, indicating that LAFLT thermal injuries do not provoke hepatic dysfunction.

The spleen plays an important part in the immune response by storing and activating immune cells.^25^ Enlargement of the spleen has been observed after burn injuries.^14^^,^^26^ This increase in spleen size relates to the systemic immune response, which correlates to the severity of the thermal injury and infiltration of immune cells to the skin during the wound-healing response.^27^ We observed that FL-treated mice had an increase in spleen size, and histology shows an expansion of the white pulp and red pulp (Figure S2). Structural changes were more evident, especially in the AFL-Medium and AFL-High treatment groups, which had splenic structure changes with partially disrupted compartmentations and disorganized white pulp, while the red pulp looked expanded with infiltration of immune cells (Figure S2).^26^

## Discussion

This study serves as proof of principle that various FL treatment modalities can temporarily stimulate metabolism in mice and induce systemic changes to body composition and neurohormonal activity. Increasing the size and density of the FL-treated area correlated with the extent of metabolic activation, demonstrated by a more pronounced and prolonged increase in basal EE, water consumption, and changes in RER. In a conventional mice burn model inflicting a 10% TBSA burn wound, Hew et al. showed a 46% peak in EE at day 4 post treatment that remained elevated for 17 days, while Yang et al. showed a 30% peak in EE at day 14 post treatment after inflicting a 30% TBSA burn wound in rats.^14^^,^^28^ Therefore, even for significantly lower TBSA thermal injury than traditional burn models, LAFLT results in a faster and higher initial EE response post treatment that last for fewer days. One of the major contributors to the increase of EE after burn injuries is wound healing.^29^ The specific characteristic of FL thermal injury is that they can be modulated in shape, size, and depth and that they result in fast and scar-free wound healing. In this study, we chose laser settings that ensured that the LAFLT thermal injury disrupted the epidermis and dermis in AFL and NAFL treatments without reaching the subcutaneous fat and the muscle layers. Mouse skin accounts for ∼14% of the body weight, therefore, a significant portion of the increased EE post-FL treatment derives from wound-healing processes involving cell proliferation, protein synthesis, and enzyme activity to repair the microthermal wounds and replace coagulated tissue.^30^^,^^31^ These processes require glucose, fat, and amino acids that are provided by hepatic gluconeogenesis, lipolysis, and auto-catabolized muscle protein.^29^ We showed that the AFL-Low treatment minimally added to the energetic requirements and only stimulated a significant EE increase for one-day post treatment, at a level close to that of the control group. This 1.2-fold increase in EE of the AFL-Low and control group is likely caused by the pretreatment, which included anesthesia, shaving, and depilation of 30% TBSA, resulting in heat loss, dermal inflammation, and stress.^32^^,^^33^^,^^34^^,^^35^ It has been estimated that heat loss due to shaving and depilation can increase metabolic demand up to 3-fold above baseline when mice are kept at room temperature.^36^ In our study, mice were kept at thermoneutrality throughout the course of the experiment, and therefore, shaving and depilation only increased the metabolic demand 1.4-fold over the baseline. In addition to the increased metabolic demand, shaving and depilation compromise the epidermal barrier causing *trans*-epidermal water loss.^34^^,^^35^^,^^37^ AFL injuries also caused damage of the epidermal barrier and increased capillary permeability, which resulted in a significant fluid loss via water evaporation and consequently resulted in an increase in water consumption of the mice.^18^^,^^38^ Additionally, the increased water uptake is a significant contributor to the observed metabolic effects after FL treatment, as it has been previously shown that an increase in water consumption has a positive effect on body weight and fat mass loss and correlates positively with EE.^32^^,^^39^ The increased EE in FL treated mice is also in part fueled by an evaporative heat loss from burn wounds.^40^ Compared to the AFL modality, NAFL treatments caused significantly smaller and shallower individual lesions, resulting in faster wound healing, reduced heat and water loss, and decreased energetic requirement. Additionally, our results indicate that compared to the AFL groups, the NAFL treatment exhibits the same positive metabolic changes in mice, including browning of adipocytes, better utilization of fats, and an overall increase in EE, while having reduced skin irritation and hypermetabolism associated side effects. In future experiments, moisturizers, medical dressings, or subcutaneous infusions could be applied to reduce the water evaporation impact.

FL treated animals also exhibited shifts in fuel source and increases in aerobic metabolism. AFL treated groups significantly decreased RER compared to controls, shifting the fuel source toward fatty acid utilization.^41^ Under resting or steady-state exercise conditions, the RER ranges from 0.7 to 1.0, however, excessive energy consumption could result in values below this range.^42^ All animals in this study exhibited a decrease in RER immediately after the intervention period, whether it was control animals or those with laser treatment. These initial changes are likely produced by many factors, including shaving, depilation and analgesic and anesthetics of large skin areas, producing alterations of the epidermal barrier properties, a factor that also influenced EE, as mentioned above.^37^ Lack of insulation is known to cause body heat loss and stimulation of thermogenesis, to maintain core temperature, even in mice kept at thermoneutrality.^36^ The acclimation period exhibited delayed recovery of the RER for those animals treated with AFL, and the delay in achieving baseline RER was correlated with the dose of AFL delivered. This suggests that the laser treatment is further inducing beta-oxidation and lipid utilization beyond the effects of the experimental setup.

The expected change in lipid stores based on higher EE and lipid catabolism was corroborated by body composition analysis showed a decrease in body weight and fat mass for all groups. Compared to the control group, however, FL-treated mice tend to have a stronger change in body and fat mass while conserving lean body mass in all except the AFL-High group. The likely contributor to the lean mass loss in the AFL-High group was the presence of scabs, which led to a decrease in locomotor activity and higher inflammatory markers. The loss of lean mass in the strongest treatment group highlights the trade-off in TBSA with the goal of promoting healthy body composition. Identifying the appropriate therapeutic window, where fat mass will be utilized without inducing inflammatory cachexia, will be an essential detail should this be considered for therapeutic purposes. One variable that is likely to be relevant to identifying this therapeutic window is baseline adiposity. Perhaps, when used on obese mice or subjects, LAFLT has the possibility of a reduced risk of muscle wasting due to excess fat. However, future studies of obese mice will have to be performed to provide in-depth information on the effectiveness and safety of LAFLT treatments.^18^^,^^43^

Altering adipocyte functionality is one approach to promoting metabolic rate. We showed that mice in all FL treatment groups had an induction of systemic browning of white adipocytes 6 days post treatment. This effect has not been previously described for FL treatment, although our study is the first of its kind to look at the effect of fractional thermal injuries at this scale of TBSA. Interestingly, a systemic browning of WAT was also observed in the AFL-Low group. These systemic effects were not expected for low FL treatment settings, since the literature shows that severe burn injuries of a minimum of ∼20% TBSA are the ones that trigger elevated noradrenaline plasma concentrations after the injury.^44^ Adrenergic stress is associated with browning white adipocytes. We did not identify elevated adrenergic activation by serum markers toward the end of the experiment, although these hormones are likely to be active closer to the intervention time. The presence of persistent browning despite low substrate for adrenergic activation suggests that non-canonical pathways associated with FL treatment are inducing brown fat biogenesis. Thus, FL treatments serve not only as a mechanism for altering body composition but also as a new tool to study the mechanisms of thermogenesis and adipocyte differentiation.

Browning of adipocytes increases lipolysis and, in burnt patients, has been linked to the development of hepatic steatosis.^45^^,^^46^ Liver damage after a severe burn injury occurs in humans and in animal models of burn injury.^14^ Post-burn liver dysfunction includes hepatocyte damage, indicated by increased levels of hepatic enzymes (ALT, AST), and substantial lipid accumulation (hepatic steatosis), among others. Although our LAFLT thermal injury model stimulated WAT browning, the livers of the treated mice did not show significant histological changes, lipid infiltration, or an increase in ALT serum levels, indicating that no hepatic damage was present on day 6 post treatment. In general, post-burn liver damage is a reversible finding that correlates to the thermal injury severity.^13^^,^^14^ However, we suspect that since fractional lesions do not extend beneath the dermis, signaling pathways that are activated by deep burns and result in fatty liver are not triggered from an inflammatory perspective. Interestingly, the extent of negative energy balance and lipid catabolism seen in the FL-treated animals would be expected to drive some hepatic lipid accumulation due to systemic lipolysis. The lack of this occurring suggests marked efficiency in lipid uptake by other organs and/or rapid flux of hepatic lipids. Whether FL treatment has a primary protective effect on hepatic lipid uptake also needs to be considered and would be a valuable goal of future investigation.

The inflammatory signature of the FL-treated animals may provide some clues into their unique metabolic phenotype. IL-6 has previously been shown to have both autocrine and endocrine effects on brown adipocytes as well as systemic signaling to promote thermogenesis and hepatic gluconeogenesis.^18^^,^^46^ We have shown that IL-6 is upregulated in FL-treated animals. While IL-6 levels have previously been shown to be chronically elevated in burn patients, we can assume that FL treatment resulted in only a temporary increase in mice since previous studies have shown that EE is strongly correlated with IL-6 levels, and we saw a gradual decline of EE over the time course of our study.^18^ Therefore, by extrapolating from EE levels that we observed, we can hypothesize that IL-6 levels likely peaked at 1–2 days post-FL treatment and decreased by day 6 post treatment when levels were measured. This would explain why significant browning of WAT can be observed for all FL-treated mice, even though only the AFL-High group shows elevated IL-6 levels on day 6 post treatment. Evidence of global immune activation does persist however, with evidence of splenomegaly at experimental completion. Previous murine studies of severe burn injury have shown that spleen changes are maximal at post burn days 7–8 caused by an increase of granulocyte-macrophages contents.^47^^,^^48^ In large burn wounds splenomegaly arises acutely and persists chronically, however, future studies will have to determine how persistent splenomegaly is post-LAFLT.

To date, this is the first study to show systemic changes, such increase in EE and adipocyte browning after FL treatment. Some of these systemic changes were even achieved for the AFL-Low group, in which laser settings were close to clinical treatments for skin rejuvenation. Therefore, studies aimed to investigate the translational potential of our mouse study are of interest. Compared to surgical and pharmacological weight loss interventions, energy-based fat removal and body sculpting alternatives such as radiofrequency, high-intensity focused ultrasound, low-level laser therapy, injection lipolysis, and cryolipolysis focus on local destruction of subcutaneous fat.^49^^,^^50^ While these devices achieve local fat reduction by non-invasive or minimally invasive destruction of subcutaneous fat, clinically no systemic effects or reduction in overall weight have been observed.^51^^,^^52^^,^^53^ Therefore, LAFLT is the first energy-based treatment that achieves global fat reduction rather than local subcutaneous fat reduction. Additional benefits of AFL and NAFL treatments are skin tightening and rejuvenation effects which also occurred in our experiments.^54^^,^^55^ While adipocyte browning lasts for at least 6 days post injury in our adult murine model, there is reason to suspect that browning of WAT may persist for several weeks or months in an adult human.^12^ While this is an estimate, there are potentially important implications for obesity treatment and for improvements in metabolism that may result from changes in adipose tissue due to LAFLT. This study is the first of its kind to highlight the specific metabolic effects that LAFLT triggers in mice, including changes in energy expenditure, body composition, adipocyte browning, and inflammatory states. This study points out that localized laser treatments can have systemic effects, which is a novel aspect of laser treatments since their intended use is historically limited to treating targets that are within the direct reach of the applied laser radiation. As hypothesized, we showed that treatment settings that correspond to treatment areas exposed in current clinical applications of the face and neck (AFL-Low) did not result in a lasting metabolic stimulation but that treatment areas that exceed current clinical applications (AFL- Medium, AFL-High, and NAFL) result in a significant metabolic stimulation. Further animal and human studies are warranted to analyze other markers of increased metabolism and to precisely ascertain the underlying mechanism of the increased metabolic rate. Finally, the ability to change metabolism in a controlled way without causing significant side effects could emerge as a promising treatment modality to increase energy expenditure and can stimulate future development of novel indications for laser treatments mimicking the effects of systemic drugs.

### Limitations of the study

The chronicity of the metabolic and inflammatory responses to FL treatment remains an important question. One limitation of this study was the duration of response, as the study period was limited to a week after the intervention. Further studies are needed to delve into the long-term safety and efficacy of this treatment modality, despite the fact that this procedure was well tolerated in the short term. The confounding aspect of hair removal also contributes to challenges in ascertaining differences in physiology between control and treated animals. Conventionally, FL treatment is applied to the skin, and hair should be avoided in order to prevent pain due to burning of the hair follicle. Future studies should also include unshaved mice as controls to determine the specific effect of shaving and depilating in our experimental settings. Additionally, the body temperature was not measured during the experiment while a drop in core body temperature could have caused some of the measured metabolic effects.

Murine modeling of FL also exhibits some limitations in the context of human translation. While mouse models of severe burn injury are known to correspond with many characteristics of the human burn response, some differences exist. For conventional burn injuries, the most relevant difference for this study is that mice only require a ˜5% TBSA burn to become hypermetabolic whereas humans require a minimum ˜20% TBSA burn.^14^^,^^15^ While we successfully stimulated the metabolic response in mice to LAFLT, humans may respond differently. Mice have a significantly higher surface area to volume ratio than humans, and this means greater heat loss for mice.^56^ Even though mice have a higher mass-specific metabolic rate compared to humans, it only partially compensates for their heat generation requirements.^56^ Therefore, mice tend to activate thermogenesis more easily than humans. Notably, at room temperature, over one-third of total energy expenditure is needed to maintain core body temperature, while for humans, a very small fraction of total energy expenditure is required.^57^ Further, mouse and human skin exhibit significant differences in their structural organization, cellular composition, and wound healing capabilities, which require careful consideration when translating findings from murine models to clinical applications in humans.^58^ Human skin possesses a thick, multilayered epidermis with a prominent stratum corneum, while mouse skin generally has a thinner, less stratified epidermis. In humans, the stratum corneum serves as a robust barrier to water loss and environmental insults, while mouse skin lacks the same degree of barrier functionality.^58^ In the area of wound healing, mouse skin primarily relies on contraction for closure, a process mediated by myofibroblasts that pull the wound edges together. Human skin, leans more toward re-epithelialization, where keratinocytes migrate over the wound bed to restore the epidermal layer, accompanied by granulation tissue formation.^58^ Furthermore, species-specific variances in growth factors, cytokines, and cellular receptors are involved in the wound healing process, adding another layer of complexity. Therefore, while murine models offer valuable insights into skin biology and wound healing, it is essential to corroborate findings in human-based systems to ensure their clinical relevance. Additionally, since human skin is much thicker than mouse skin and wound healing rates differ, the laser energy per pulse must be adjusted accordingly for clinical use.

## STAR★Methods

### Key resources table

**Table:** 

REAGENT or RESOURCE	SOURCE	IDENTIFIER
**Antibodies**		

Rabbit polyclonal anti-Ucp1	Abcam	Cat#10983
SignalStain Boost IHC detection reagent	Cell Signaling	Cat#8114

**Chemicals, peptides, and recombinant proteins**		

NP-40	ThermoScientifc	Cat#28324
OCT compound Tissue-Tek	FisherScientific	12351753
Oil Red O	Sigma-Aldrich	O0625
SignalStain DAB substrate kit	Cell Signaling	Cat#8059
Nitro blue tetrazolium chloride	ThermoScientifc	Cat# 34035

**Critical commercial assays**		

Noradrenaline high sensitive ELISA	Eagles Biosciences	Cat# NOU39-K01 0
Quantikine ELISA Mouse Il-6	R&D Systems	Cat#NOU39-K01 0
Alanine aminotransferase activity kit	Sigma-Aldrich	Cat#MAK052
Triglyceride assay kit	Abcam	Cat#Ab65336
Trichrome Masson, fast green stain kit	NewComerSupply	Cat#9180A
Periodic acid Schiff (PAS) stain kit	NewComerSupply	Cat#9162

**Experimental models: Organisms/strains**		

Mouse: C57BL/6J	The Jackson Laboratory	JAX: 000664

**Software and algorithms**		

GraphPad Prism Version 9	GraphPad	https://www.graphpad.com/ RRID:SCR_002798
Microsoft Excel	Microsoft	https://www.microsoft.com RRID:SCR_016137
ImageJ	National Institute of Health	https://imagej.nih.gov/ij/

**Other**		

AFL Ultrapulse 10600 nm CO_2_ laser	Lumenis	https://lumenis.com/aesthetics/products/ultrapulse/
NAFL Icon 1540 nm Er:glass laser	Cynosure	https://www.cynosure.com/product/palomar-icon-aesthetic-system/
Promethion respiratory system	Sable Systems International	https://www.sablesys.com/products/promethion-core-line/
Nanozoomer S60	Hamamatsu	C13210-01

### Resource availability

#### Lead contact

Further information and requests for resources should be directed to the lead contact, Dr. Dieter Manstein (dmanstein@mgh.harvard.edu).

#### Materials availability

This study did not generate new unique reagents.

#### Data and code availability

(1) All data reported in this paper will be shared by the lead contact upon request.
(2) This paper does not report original code.
(3) Any additional information required to reanalyze the data reported in this paper is available from the lead contact upon request.

### Experimental model and study participant details

The animal protocol was approved by the Ethics Committee for Animal Care, the Subcommittee on Research Animal Care, and the Institutional Animal Care and Use Committee of Massachusetts General Hospital. 22-week-old wild-type male C57BL/6J mice (Jackson Laboratory, Bar Harbor, ME, USA) were kept single-housed with *ad libitum* access to standard laboratory chow (Formulab 5008, 3.56 kcal/g, LabDiet, USA) and water. Males were selected for this study since females are more variable than males due to their cyclical reproductive hormones effect. Additionally, males show better metabolic changes than females.^59^^,^^60^

### Method details

#### FL treatments

Mice were treated using an AFL Ultrapulse 10600 nm CO_2_ laser with DeepFX handpiece (Lumenis, Yorkneam, Israel) and a NAFL Icon 1540 nm Er:glass laser (Cynosure, Westford, MA, USA). The CO_2_ AFL generated 1 cm^2^ fractional arrays at 10% (289 MTZs/cm^2^) or 15% (441 MTZs/cm^2^) density settings. Histological analysis shows that the fractional density setting describes the ablation hole density while neglecting the coagulation zone (CZ). Therefore, a 10% fractional density setting resulted in lesions (ablation + CZ) that covered 16% of the treatment area and a 15% fractional density setting resulted in lesions (ablation + CZ) that covered 25% of the treatment area.

The body surface area of each mouse was determined by the Meeh equation: TBSA = kW^2/3^, where TBSA is total body surface area, W is weight, and k is an empirically determined constant.^61^ The Meeh’s constant for C57BL/6J is k = 10, which resulted in TBSAs of 91–114 cm^2^.^62^

Prior to anesthesia, sustained-release buprenorphine (1 mg/kg body weight) was administered by subcutaneous injection to all mice. Sustained-release buprenorphine (Zoopharm, Laramie, WY) alleviates post-procedural pain for a minimum duration of 72 h. Mice were anesthetized by intraperitoneal injection of ketamine (90 mg/kg body weight) and xylazine (9 mg/kg body weight). The backs of the mice were then shaved and depilated with hair removal cream (Nair, Church & Dwight, USA). Control mice underwent the same experimental procedures except for the fractional laser treatment. Mice were treated in 3 AFL groups with different preset treatment areas: AFL-Low, AFL-Medium, and AFL-High. For the AFL-Low setting, 10% of mice TBSA were treated at 10% fractional density laser setting, resulting in a coverage rate of thermal injury of 1.6% TBSA. For the AFL-Medium setting, 20% of mice TBSA were treated at 10% fractional density setting, resulting in a coverage rate of thermal injury of 3.2% TBSA. For the AFL-High setting, 32% of mice TBSA were treated at 15% fractional density setting, resulting in a coverage rate of thermal injury of 8% TBSA. The CO_2_ laser was operated at 17 mJ/pulse with pulse durations of 1 ms for all AFL settings. The NAFL, in combination with a 15 mm lens handpiece (Cynosure, Westford, MA, USA), generated 1.8 cm^2^ fractional arrays with 2.2% fractional density (320 coagulation zones/cm^2^). Histological analysis shows that the fractional density agrees with the NAFL settings. For the NAFL group, 25% of mice TBSA were treated at 15% fractional density (8 x passes), resulting in a coverage rate of thermal injury of 4% TBSA. The Er:glass laser was operated at 8 mJ/pulse with a pulse duration of 10 ms. After the laser treatment, exposure areas were covered in petrolatum to prevent the wounds from drying out.

#### Metabolism and body composition

Five groups (Control n = 9, AFL-Low n = 8, AFL-Medium n = 8, AFL-High n = 7, NAFL n = 7) of mice were individually housed for 1 day before being placed in metabolic cages. After an acclimation period of 2 days inside the metabolic cages, 1-day pre and 6 days post-laser treatment were recorded. Indirect calorimetry measurements were collected using two 8-cage Promethion respiratory systems (Sable Systems International (SSI), Las Vegas, NV, USA). In the system, rates of oxygen consumption (VO_2_) and carbon dioxide production (VCO_2_) were acquired by indirect calorimetry with a sampling frequency of 1 s. Respirometry values were determined every 5 min. Food intake, water intake, locomotor activity, and body mass were recorded continuously by gravimetric measurements within the cages. Locomotor activity was determined according to beam breaks within a grid of infrared sensors built into each cage. Energy expenditure was calculated using the Weir equation (Energy expenditure = 3.941 kcal/L x VO_2_ + 1.106 kcal/L x VCO_2_).^63^ Energy expenditure, activity, and food and water consumption are displayed as the average per specified periods of time. During the experiment, mice were continuously provided food and water and exposed to a day/night cycle of 12 h/12 h under controlled temperature (29°C) and humidity (40–60%). The respiratory exchange ratio (RER = VCO_2_/VO_2_) is measured by the amount of CO_2_ produced and O_2_ consumed by the animal. RER values of ∼0.7 indicate that fats are the primary fuel source in the body, whereas RER values of ∼1.0 indicate that carbohydrates are the primary fuel source.^42^ Lean and fat mass were measured by quantitative magnetic resonance using an EchoMRI-100 (EchoMRI LLC, Houston, USA) without anesthesia and according to the manufacturers’ instructions, immediately before laser treatment, and 6 days after treatment.

#### Noradrenaline and interleukin-6 determination

Mouse Elisa kits were used to determine noradrenaline (Eagles Biosciences, Amherst, NH, USA) and IL-6 levels (R&D Systems, Minneapolis, MN, USA), per manufacturers’ instructions.

#### Alanine aminotransferase activity (ALT) quantification

A commercial kit was used to determine alanine aminotransferase activity (Sigma MAK052, Sigma-Aldrich St. Louis, MO, USA) in serum, per the manufacturer’s instructions.

#### Triglyceride quantification

A quantitative triglyceride (TG) assay kit (ab65336, Abcam, Cambridge, USA) was used. Liver tissue was resuspended and homogenized in 5% nonionic detergent (NP40, ThermoFisher, Waltham, MA, USA). After heating to 90°C, insoluble material was eliminated and mixed with lipase to convert TG to fatty acid. After mixing with reaction buffer, samples were read at 570 nm, and concentrations were determined using a standard curve.

#### Histological and wound analysis

Sections of skin, livers, spleens, and fat pads were dissected from mice on day 6 after laser treatments. Fat pad dissections consisted of different white adipose tissue (WAT) pads: inguinal (Ing), epididymal (Epi), retroperitoneal (Rp), and anterior subcutaneous (As). Parts of the skin, liver, and spleen were fixed in 10% formalin, embedded in paraffin, transverse cut (5 μm), and processed with hematoxylin and eosin (H&E) staining. Sections of fat pads were incubated with U CP1 antibody (Abcam, Waltham, MA, USA) followed by a secondary antibody conjugated to horseradish peroxidase (Boost IHC detection reagent) and by 3,3′-Diaminobenzidine substrate (SignalStain DAB substrate). Livers were additionally embedded in optimal cutting temperature compound (Tissue-Tek OCT, Sakura Finetek, USA), and 20 μm thick vertical sections were obtained by serial cryo-sectioning. Sections were stained with a lipid stain (Oil Red O, Sigma-Aldrich, St. Louis, MO, USA) and analyzed using a digital slide scanner (NanoZoomer S60; Hamamatsu) at 40× magnification. Additional stains were performed for the liver to assess collagen deposition and glycogen using the Masson Trichrome staining kit and PAS diastase staining kit (PAS-D), respectively, following manufacturers’ instructions (New customer supply, Middleton, WI, USA). Wounds were monitored with photographs using a digital camera (D60, Nikon, Tokyo, JP) immediately before and after laser treatment and on day 6 immediately after euthanasia (Figure 4).

### Quantification and statistical analysis

Data analyses and graphs were generated in Excel. GraphPad Prism 9.0 was used for statistical analyses. Metabolic cage and body composition results of pre- and post-treatment data were analyzed by paired t-tests. To analyze body composition changes and quantify organ and blood samples, the Shapiro-Wilks test was used to check for normality. Normally distributed data was analyzed by a one-way ANOVA, followed by a Dunnett’s post-hoc test. Non-parametric data was analyzed by the Kruskal-Wallis test followed by a Dunn’s post-hoc test. For daily analysis of energy expenditure, RER, and water consumption, the Shapiro-Wilks test was used to check for normality and then a non-parametric repeated measures ANOVA and Friedman post-hoc test was performed.
