# Supplementary material for: Large area fractional laser treatment of mouse skin increases energy expenditure
Source: iScience. 2023 Dec 7;27(1):108677. doi: 10.1016/j.isci.2023.108677 (PMC10783634; doi:10.1016/j.isci.2023.108677)
Supplement: Document S1. Figures S1–S3 and Table S1 [file mmc1.pdf]

iScience, Volume *27*

Supplemental information

Large area fractional laser treatment of mouse skin increases energy expenditure

Nunciada Salma, Michael Wang-Evers, Daniel Karasik, Armen Yerevanian, Heather Downs, Tuanlian Luo, Abigail E. Doyle, Zeina Tannous, Jose M. Cacicedo, and Dieter Manstein

Supplementary Information

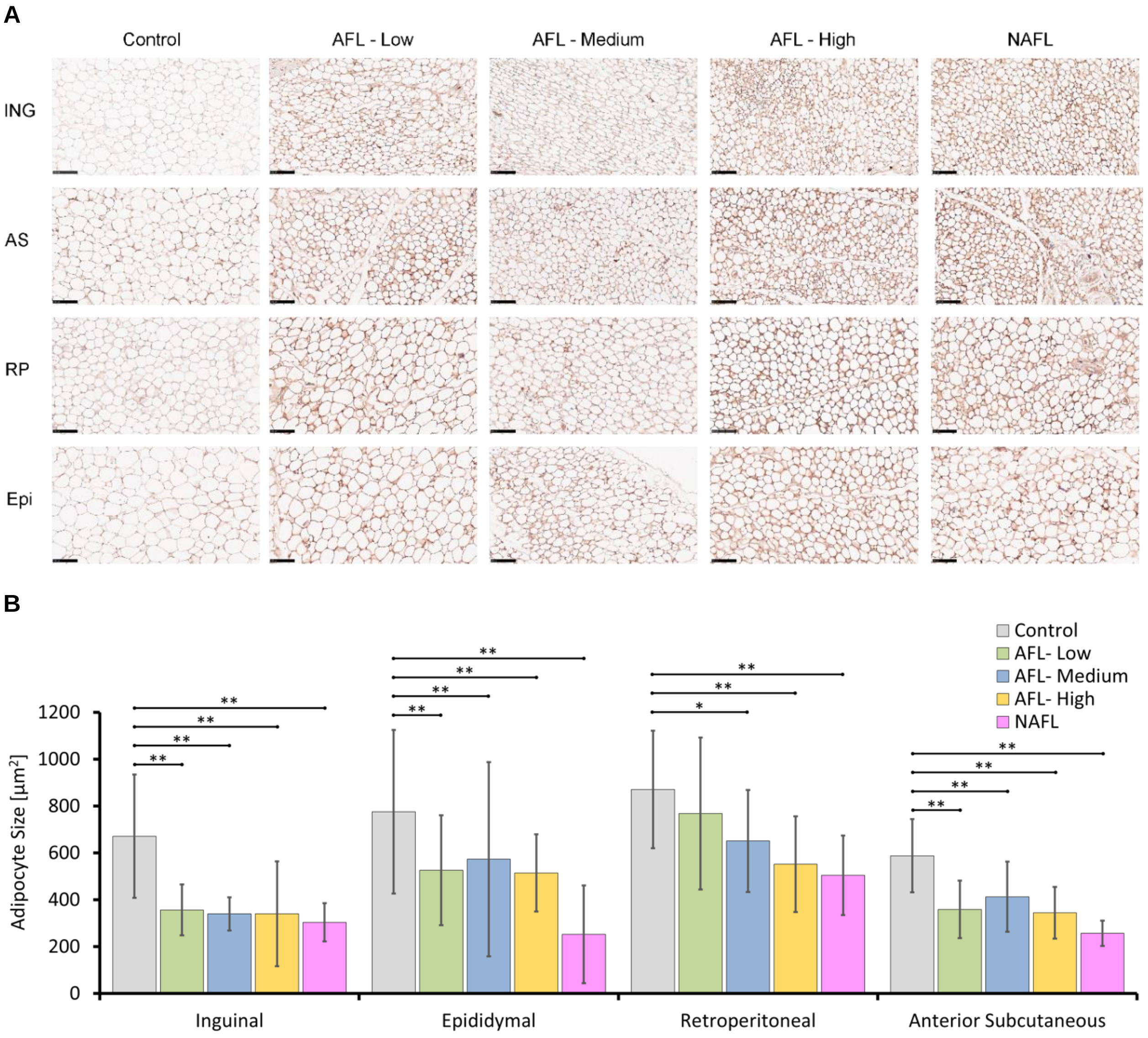

Figure S1. Analysis of browning and size of adipocytes, related to Fig. 6. A) FL treatment induced adipose tissue browning. UCP1 immunostaining of inguinal, epidydimal, retroperitoneal, and anterior subcutaneous fat pads from control mice compared to FL treated mice (6 days post-treatment). Representative images from n =3-5 mice per group. Multilocular UCP1-positive adipocytes are distinguished in all FL-treated mice compared to control mice. **B)** Analysis of the average size of adipocytes of several fat pads. A minimum of 650 adipocytes were automatically analyzed for each group and fat pad. Data are represented as mean ± SD. Statistical significance was analyzed by t-test, *p < 0.05, p**<0.001.

Adipocyte size analysis was performed in ImageJ using the following macro algorithm:

```
run("Images to Stack", "use");
run("8-bit");
setAutoThreshold("Default dark");
//run("Threshold...");
setThreshold(235, 255, "raw");
//setThreshold(235, 255);
setOption("BlackBackground", false);
run("Convert to Mask", "method=Default background=Dark list create");
run("Options...", "iterations=1 count=1 pad do=Nothing");
run("Erode", "stack");
run("Analyze Particles...", "size=70-20000 circularity=0.25-1.00 show=Overlay display exclude summarize stack");
```

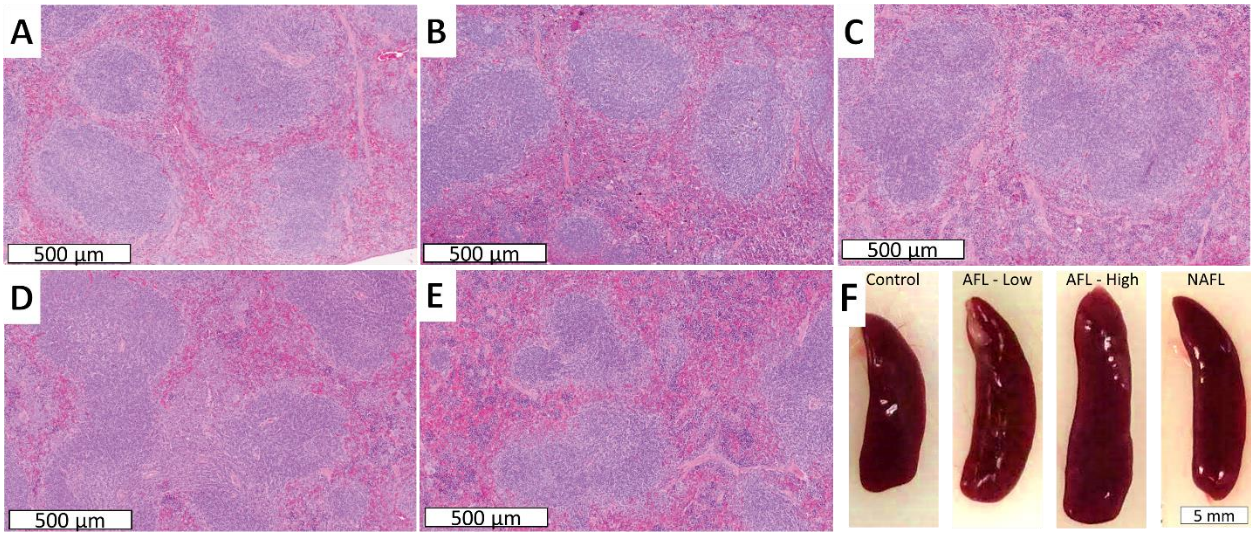

Figure S2. Spleen histology, related to Fig. 7. Representative histology of H&E-staining of the spleens of **A)** control, **B)** AFL-Low, **C)** AFL-Medium, **D)** AFL-High, and **E)** NAFL treatment groups. White pulp lymphoid tissue (purple color) and red pulp connective tissue (pink color) can clearly be distinguished. **F)** Photographs of the spleen showing enlargement after laser treatment.

The increase in spleen size is related to the degree and density of the thermal skin damage, and was more evident in the AFL groups, with a minor degree in the NAFL group. AFL groups, particularly the AFL-Medium and AFL-High groups, showed a noticeable infiltration of immune cells.

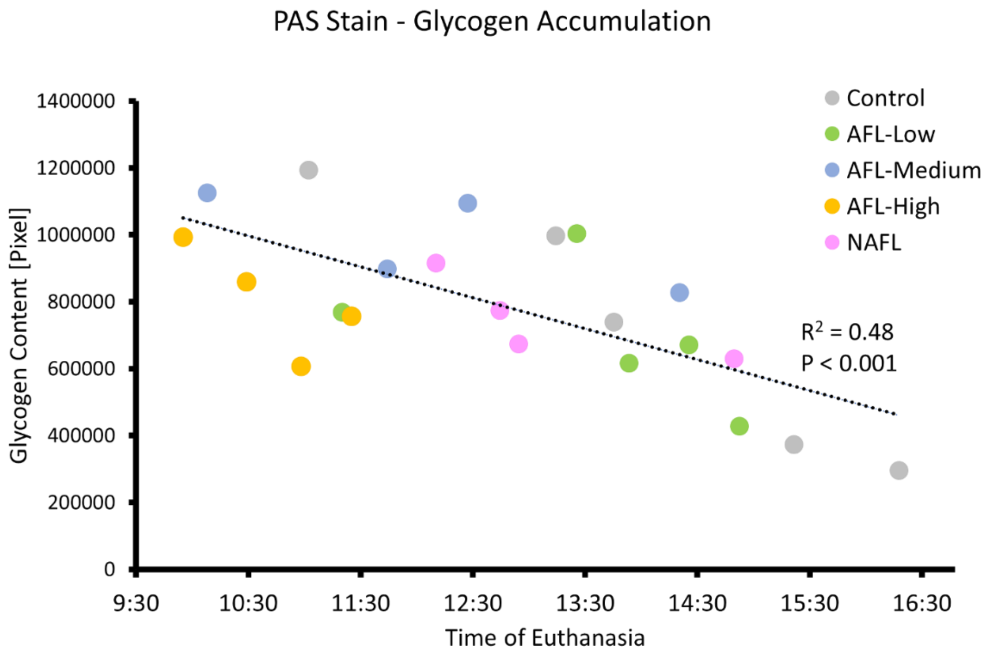

Figure S3. Glycogen accumulation, related to Fig. 7. Glycogen accumulation of the liver correlated with the time of animal euthanasia. A PAS Schiff stain was used to highlight glycogen which was then analyzed by using ImageJ. The R^2 value shows how close the data is to the calculated fitted regression line which presented a significance of $p < 0.001$.

Glycogen accumulation analysis was performed in ImageJ using the “Color Threshold” and “Measure” function.

	# Mice per Group	# Mice for Metabolic Analysis	# Mice for Tissue/Organ Analysis
Control	9	9	9
AFL-Low	9	8	8
AFL-Medium	9	8	8
AFL-High	9	7	7
NAFL	9	6	8

Table S1. Mice distribution, related to Fig. 1. An equal sample size of 9 mice per experimental group was anticipated. For organ and tissue analysis 5 mice were excluded due to the following criteria: 4 mice fell below the minimum starting weight criterion of 25g for inclusion in the study and one mouse presented a significant leg wound unrelated to the laser treatment. An additional 2 mice were excluded from the metabolic cage analysis due to technical difficulties which adversely affected data collection.
